# Meta-analysis of microRNAs expression in head and neck cancer: uncovering association with outcome and mechanisms

**DOI:** 10.18632/oncotarget.19224

**Published:** 2017-07-13

**Authors:** Joshua Lubov, Mariana Maschietto, Iman Ibrahim, Alex Mlynarek, Michael Hier, Luiz Paulo Kowalski, Moulay A. Alaoui-Jamali, Sabrina Daniela da Silva

**Affiliations:** ^1^ Department of Otolaryngology Head and Neck Surgery, Segal Cancer Centre and Lady Davis Institute for Medical Research, Sir Mortimer B. Davis-Jewish General Hospital, Departments of Medicine and Oncology, McGill University, Montreal, QC, Canada; ^2^ Brazilian Biosciences National Laboratory, National Center for Research in Energy and Materials, Campinas, SP, Brazil; ^3^ Department of Head and Neck Surgery and Otorhinolaryngology, AC Camargo Cancer Center, São Paulo, SP, Brazil

**Keywords:** miRNAs, prognosis, head and neck cancer, therapeutics

## Abstract

Head and neck squamous cell carcinoma (HNSCC) is often diagnosed at advanced stages, incurring significant high mortality and morbidity. This review explored the risk stratification of miRNAs, and investigated the impact of miRNA networking in HNSCC prognostication. We performed a meta-analysis and a systematic literature search on online databases for papers published prior to December 1, 2016. The list of miRNAs was uploaded to MetacoreTM to construct a protein-protein interaction network, which was used to identify targets of the miRNAs and potential drugs. In addition, a representative network was further validated by immunohistochemistry in a cohort of 100 patients. We found 116 studies that included 8,194 subjects, in which the relationship between miRNA expression and prognosis of HNSCC were analyzed. Significant elevated expressions of 27 miRNAs and decreased expression of 26 miRNAs were associated with poor outcome. After excluding the studies causing heterogeneity, a fixed model was applied, which showed a statistically significant association between increased expression of miR-21 and poor survival (Pooled HR = 1.81,95% CI = 0.66–2.95, *P* < 0.005). We identified four networks affected by the miRNAs expression and enriched in genes related to metabolic processes and regulation of cell mitogenesis in response to extracellular stimuli. One network point out to 16 miRNAs directly or indirectly involved in the regulation of androgen-receptor (AR). Evaluation of AR protein expression in our cohort revealed that patients with upregulation of AR had poor survival rates (log-rank test, *P* < 0.005). This study showed that miRNAs have potential prognostic value to serve as screening tool for HNSCC during the follow-up. In addition, the implementation of a network-based analysis may reveal proteins with potential to be used as a biomarker.

## INTRODUCTION

Head and neck squamous cell carcinoma (HNSCC) consists of a heterogeneous group of malignancies arising from the oral cavity, nasal cavity, paranasal sinuses, pharynx, and larynx. HNSCC is the sixth most common cancer and the seventh cause of cancer-related deaths worldwide [1, 2]. The incidence rate is higher in developed countries and the majority of patients present with advanced stages at diagnosis, characterized by local aggressiveness and high potential for regional and distant metastasis [2, 3]. Despite the considerable progress in surgery, radiotherapy, and chemotherapy over the last few decades, the survival rates have improved only marginally, and the overall 5-year survival probability for patients with HNSCC is among the lowest of the major cancers, such as breast and prostate [4, 5]. The high mortality and poor prognosis are associated with frequent distant metastasis at initial diagnosis, as well as a high incidence of inoperable local and regional relapses after initial treatment [6].

MicroRNAs (miRNAs), a class of mature, non-coding, single-stranded RNAs with 21–23 nucleotides, were proposed as promising biomarkers for patients with cancer diagnosis and follow-up [7]. With the capacity of targeting hundreds of genes, miRNAs display a role in virtually all cellular pathways, with a critical impact in a variety of biological processes, including proliferation, metabolism, and apoptosis [6]. The more recent miRBase database release (version 21) contains 1,881 precursors and 2,588 mature miRNAs in humans [8]. Although there is an increasing number of studies profiling miRNAs and exploring their relationship to tumor development and progression, the inter-lab reproducibility of the results is often problematic due to the small sample size, as well as to biological variations and non-standardized assays for miRNA detection [9].

Previous studies have found deregulation in expression of miRNAs in HNSCC and explored their use as potential biomarkers for cancer detection and/or prognosis. For instance, studies in HNSCC have shown that miR-19, miR-21, and miR-375 are associated with poor survival probability in laryngeal squamous cell carcinoma [10–12]. The prognostic value of a signature based on six miRNAs expression (let-7c, miR-125b, miR-129, miR-337, miR-654, miR-99a) was proposed to discriminate high- *versus* low-risk patients with oropharyngeal squamous cell carcinoma [13]. Here, we systematically reviewed all articles investigating the prognostic value of miRNA expression HNSCC patients. The meta-analysis was done to confirm the clinical relevance of the most investigated microRNA in HNSCC. Then, we used a network-based analysis to prioritize putative molecular targets of existing drugs to open new avenues for further experimental studies in HNSCC.

## MATERIALS AND METHODS

### Search of publications

**Figure 1:**
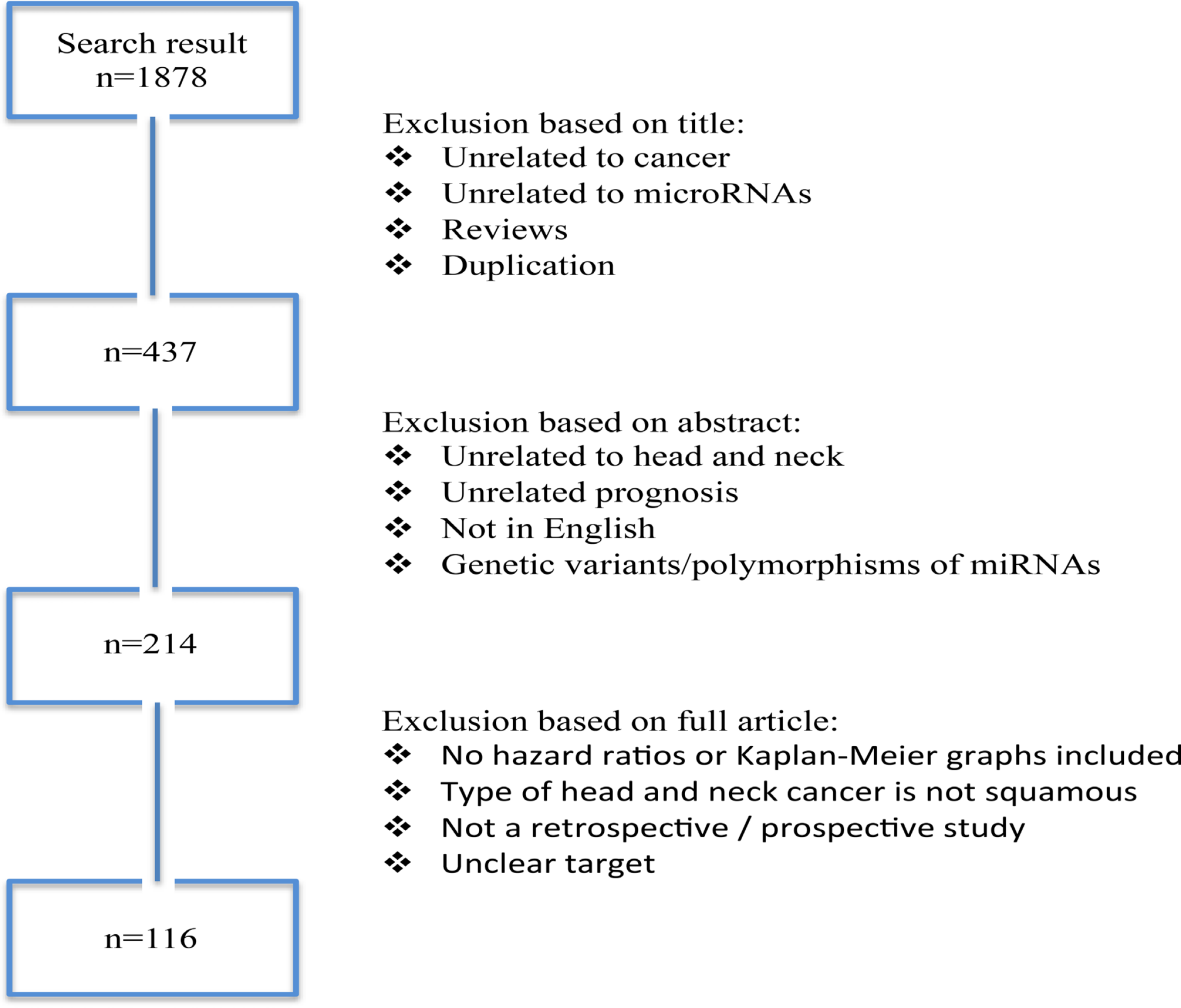
Flow diagram of search and study selection process Following the guidelines of the Meta-analysis of Observational Studies in Epidemiology group (MOOSE), we performed a broad and sensitive search on online databases to identify the studies that examined associations between different microRNAs expression and HNSCC prognosis. A systematic literature search for relevant studies up to December 2016. In this study, we considered the clinical endpoints overall survival (OS) and disease specific survival (DFS) as acceptable outcomes. The prognostic value was demonstrated using hazard ratio (HR) with 95% confidence interval (CI).

We conducted a systematic literature search of PubMed, Wiley Online Library, EMBASE, Web of Science, Scopus, and Cochrane databases between 2008 and December 1, 2016, for studies that analyzed associations between miRNAs expression, HNSCC prognosis, and predictive impact. We used the key words including miRNAs truncations, abbreviations, synonyms, and subsets for the strategy search: “head and neck neoplasms” or “facial neoplasms” or “head and neck cancer” or “oral cancer” or “head and neck squamous cell carcinoma” or “HNSCC” or “tongue cancer” and “microRNAs” or “miRNA” or “miRs” or “miR-*” and “prognostic” or “prognosis” or “predictive”). Searches in Gene Expression Omnibus (GEO, www.ncbi.nlm.nih.gov/geo/) and ArrayExpress (www.ebi.ac.uk/arrayexpress) repositories were also performed. We designed our strategy to be optimized for a sensitive and broad search (Figure 1). Two librarian experts in systematic review methods hand searched the references list to find additional articles.

### Inclusion and exclusion criteria

The title and the abstract of all potentially relevant studies were evaluated for their contents before the retrieval of full articles. Full text of each study was carefully evaluated. Studies were required to meet the following inclusion criteria to be eligible: 1) included human case-control studies; 2) included clinical studies related to the prognostic value of miRNAs in HNSCC; 3) the studies made available information on true positives, false positives, false negatives, and true negatives; 4) publications were not duplications; and 5) studies were not in the form of abstracts or editorial articles. Survival outcome was further explored considering Hazard ratio (HR) with confidence of interval and HR with *P*-value or Kaplan-Meier graph.

The exclusion criteria involved non-English papers, case reports, letters, and reviews. We also excluded cross-sectional studies that addressed associations with tumor stage, tumor histology, tumor size, tumor differentiation, or malignant potential without specifically examining associations with clinical outcome. Expression studies of individual preselected candidate miRNAs or studies using only cell lines were excluded. Papers that fulfilled the criteria were processed for data extraction and the disagreements were resolved by discussion.

### Data extraction and quality assessment

The quality of all the included studies was systematically assessed according to the Dutch Cochrane Centre for epidemiological studies including: a) clear definition of outcome assessment by representing it in overall survival (OS) or disease-free survival (DFS) or disease specific survival (DSS); b) clear definition of the assay used for the measurement of miRNA (e.g. quantitative real time polymerase chain reaction (qRT-PCR), *in situ* hybridization (ISH); c) clear definition of cut-off, d) at least two year of follow-up; d) definition of the anatomical site; e) definition of the target population (country where the study took place). To be qualified, all the criteria had to be mentioned in the manuscript; otherwise the study was excluded from the systematic review.

Data were extracted from for final eligible articles as follows: first author, year of publication, impact factor of the journal publication, the country of origin, study design, population studied, subjects’ ethnicity, the number of cases and controls, cancer types, source of control, miRNA profiling expression, specimen, anatomic location, survival analysis (clinical endpoint), and follow-up. The methodological qualities of the selected eligible articles were assessed by the Quality Assessment of Diagnostic Accuracy Studies 2 (QUADAS-2) score system. The QUADAS-2 tool combines the index of patient selection, the index test, the reference standard, and flow and timing to evaluate risk of bias and applicability concerns.

### Study population

A retrospective study was performed by analyzing data from 100 patients with primary HNSCC diagnosed and treated at the Department of Head and Neck Surgery and Otorhinolaryngology, A.C. Camargo Cancer Center, Sao Paulo, Brazil (Supplementary Table 1). The eligibility criteria included previously untreated patients with diagnosis of HNSCC submitted to treatment in the same institution. This study was carried out with the approval of the Human Research Ethics Committee (CEP # 875/07).

### Immunohistochemistry (IHC) analysis

The incubations with the primary antibodies anti-androgen receptor (AR) (Dako, 1:100) diluted in PBS were made overnight at 4°C. Positive and negative controls were included in all reactions. IHC reactions were performed in duplicate on different tissue levels. IHC scoring was blinded to the outcome and clinical aspects of the patients. The percentage of AR positive nuclei was calculated with an image computer analyzer (Kontron 400, Carl Zeiss, Germany).

### Data analysis

The statistical analyses were performed using the STATA 12.0 statistical software. The pooled parameters sensitivity, specificity, diagnostic hazard ratio (HR), and their 95% CIs were calculated to evaluate the overall diagnostic accuracy. For each dataset, miRNA expression was processed as following: at first, the ambiguous probe sets (mapped in more than one gene) were filtered out; then, differential expression analysis of the probes sets between the compared samples (case-control/better-worse prognosis) was conducted (*t* test, two-tails, unequal variation); for each miRNA the probe with the most significant *P*-value was selected and the probe set's expression level was assigned as the miRNA's expression level. The reason to assign gene expression in this way is that we assume that miRNA expression has significantly changed between the compared groups (good *versus* poor prognosis). Thus, the expression of the probe set with the most significant *P*-value between compared groups was considered the best candidate to represent the expression of the miRNA. Statistical analysis considered the weighted effect and the effect size was adjusted.

### Network and enrichment analyses

The list of miRNAs was submitted to Metacore^TM^ (http://thomsonreuters.com/metacore/) looking for enriched signaling cellular pathways (KEGG) and gene ontology (GO) besides retrieving target genes under a gene-gene interaction network perspective. An enrichment analysis for GO was performed for networks with *p* < 10^–30^ and Z-score > 90. In addition, we searched for drugs acting in the genes from these networks.

## RESULTS

### Overview of the included studies

According to described search criteria, we identified 1,878 articles. After exclusion of duplication, unrelated to cancer or miRNAs, and reviews, there were 437 articles. Additional 223 studies were excluded, as they were either abstracts or irrelevant studies regarding prognostic impact in HNSCC, leaving 214 studies for further full-text examination. Titles and abstracts retrieved through this search were screened by three of the authors and after a careful reading of the texts, 98 studies without enough data were removed. Finally, we had 116 studies involving 8,194 subjects analyzed the relationship between miRNA expression and prognosis of HNSCC, from which data were extracted (Figure 1). A short description of the studies and the acronyms by which the studies were referred to is provided in Supplementary Table 2. The QUADAS-2 evaluation reported that all studies had moderately high scores, indicating a relatively high quality of the studies included in our review. The median impact factor was 3.8 (range: 0.672 to 9.329).

Of the 116 articles exploring miRNA and prognostic impact in HNSCC, 88 focused on Asian populations, while the remaining 28 articles recruited Caucasian participants. The expression levels of miRNA were widely analyzed by qRT-PCR in 113 (97%) studies. The remaining used ISH or FISH as method. The anatomic location was predominantly the oral cavity (*n* = 62), followed by larynx (*n* = 19), nasopharynx (*n* = 19), mixed sites (*n* = 14 – including nasopharynx, oropharynx, paranasal sinuses, maxillary sinus, oral cavity, and hypopharynx), and hypopharynx (*n* = 2). A total of 98 of the 116 articles measured single miRNA expression levels, while the remaining 18 investigated the expression of multiple miRNAs. Among these articles, eight and six measured miRNA expression levels in plasma and serum, respectively. These were excluded from further analyses. A detailed screening showed that 82 of 116 studies (Supplementary Table 1) did not report hazard ratio (HR). Therefore, they were excluded during the discussion of our systematic review for inadequate or unrelated reporting of prognostic criteria, which then resulted in 34 remaining studies with complete information (Table 1) [14–47].

**Table 1:** Hazard ratio and expression of the miRNAs associated with poor prognosis

Author	miR	HR (OS)	HR (DFS)	CI	*p*-value	Expression associates with worse prognosis (low/high)	Follow-up (months)
Alajez et al. 2011	218	2.4	−	−	0.04	Low	86.4
Avissar et al. 2009	21	**1.68**	−	1.04–2.77	0.034	High	60
Chang et al. 2013	17	2.47	−	1.37–4.44	0.016	Low	60
	21a	3.44	−	1.45–8.15	0.001	Low	60
Childs et al. 2009	205	**2.51**	**2.93**	1.12–5.61	0.025 (OS); 0.008 (DFS)	Low	˜60
	Let-7d	**1.73**	**2.3**	1.16–4.56	0.166 (OS); 0.017 (DFS)	Low	˜60
Ganci et al. 2014	21-3p	−	4.2	1.1–15.98	0.03	High	60
	96-5p	−	5.7	1.52–21.3	0.002	High	60
	130b-3p	−	2.9	0.8–11	0.02	High	60
	141-3p	−	4	1.26–13.9	0.04	High	60
Gee et al. 2010	210	6.88	−	2.30–20.53	0.008	High	53
Harris et al. 2012	375	**12.8**	−	3.17–51.73	< 0.05	Low	72
Jia et al. 2014	26a	0.216	−	0.064–0.725	0.013	Low	48
Jia et al. 2013	195	0.322	−	0.120–0.865	0.006	Low	48
Jung et al. 2012	21	5.31	−	1.39–20.38	0.015	High	< 180
Ko et al. 2014	21	2.97	−	1.34–6.59	0.007	High	˜200
Li et al. 2009	21	**1.027**	−	1.018–1.04	0.008	High	˜70
Liao et al. 2015	1246	2.82	−	1.07–7.43	0.036	High	60
Lin et al. 2014	206	6.245	−	−	0.015	High	72
Liu et al. 2013	196a	−	**2.57**	1.20–5.48	0.02	High	60
Liu et al. 2013	451	1.98	1.68	1.16–3.34 (OS); 1.07–2.62 (DFS)	0.01 (OS); 0.02 (DFS)	Low	96
Liu et al. 2014	134	−	**2.17**	1.17–5.12	0.01	High	60
Luo et al. 2013	18a	2.41	−	1.28–4.53	0.006	High	˜60
Ni et al. 2015	143	**7.332**	−	−	0.002	Low	72
Ogawa et al. 2012	34a	−	**200**	0.29–3.44	0.0019	Low	53
Peng et al. 2014	Let-7g	−	3.267	1.164–9.174	0.025	Low	60
Re et al. 2015	34c-5p	−	3.05	1.44–5.38	0.001	Low	˜60
Sasahira et al. 2012	126a	−	**2.631**	0.989–7.985	0.048	Low	˜60
Shen et al. 2012	34a	−	4.101	0.269–60.24	0.043	Low	36
Shi et al. 2015	155	**6.986**	-	1.684–28.997	0.0002	High	50
Tian et al. 2014	203	3.3482	-	1.5287–7.3333	0.0022	Low	60
Tu et al. 2015	372	−	**2.57**	1.20–5.48	0.002	High	60
	373	−	**2.62**	1.47–4.64	0.001	High	60
Wu et al. 2014	218	**2.51**	−	1.32–4.77	0.005	Low	60
Wu et al. 2014	9	**3.18**	−	2.19–11.91	0.014	High	60
Wu et al. 2013	Lin28B	**1.473**	−	1.057–2.053	0.022	High	˜100
Wu et al. 2014	19a	2.26	−	−	0.034	High	˜77
Xu et al. 2013	153	2.295	−	1.168–4.508	0.0269	Low	˜60
	200c	2.202	−	1.110–4.371	0.0369	Low	˜60
Zeng et al. 2012	20a	5.682	−	1.992–16.206	0.01	High	˜33
Zhang et al. 2015	23a	**6.712**	−	2.076–21.700	0.03	High	60

Bold = Adjusted Hazard Ratio.

### miRNAs associated with poor prognosis in HNSCC

**Figure 2:**
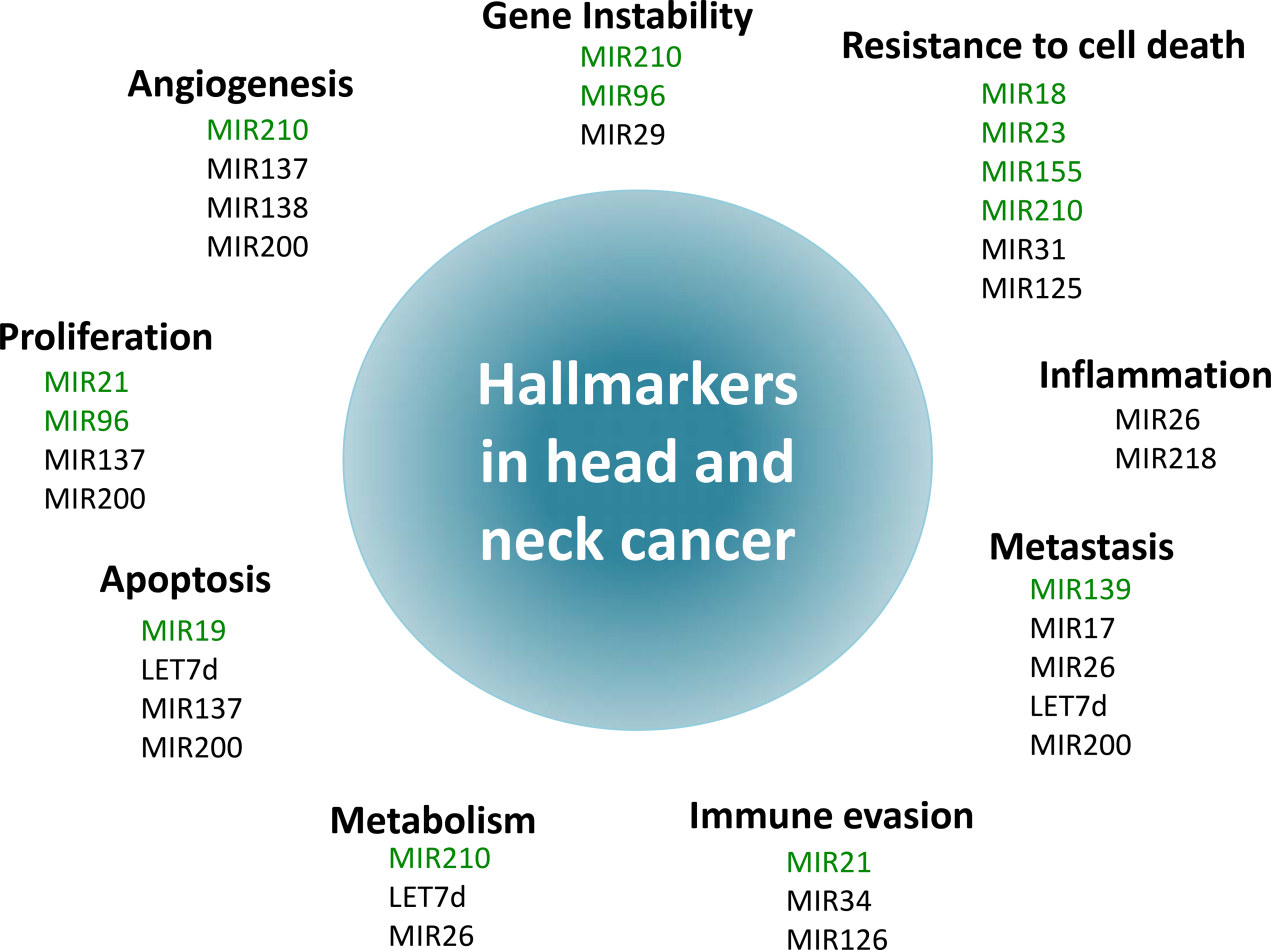
Schematic illustration of the association between microRNAs and the hallmarks of head and neck cancer The hallmarks constitute an organizing principle for rationalizing the complexities of neoplastic disease [72, 73]. Ten hallmarkers of cancer were considered: Sustaining proliferative signaling, evading growth suppressors, resisting cell death, enabling replicative immortality, inducing angiogenesis, and activating invasion and metastasis, deregulation of cellular energetics, avoidance of immune destruction, genome instability and mutations and tumor promoting inflammation. Each hallmark shows some examples of microRNAs that influence the particular cellular function in HNSCC. Of note, some microRNAs influence more than one hallmark indicating to the multiple pathways regulated by them.

Our findings reveal that significant elevated expressions of miR-7, miR-9, miR-15, miR-18, miR-19, miR-21, miR-23, miR-24, miR-93, miR-96, miR-99, miR-130, miR-139, miR-141, miR-155, miR-181, miR-195, miR-196, miR-210, miR-211, miR-214, miR-222, miR-296, miR-302, miR-331, miR-345, and miR-424 were associated with poor prognosis in HNSCC. Conversely, decreased expressions of miR-17, miR-26, miR-29, miR-31, miR-34, miR-125, miR-126, miR-137, miR-138, miR-143, miR-152, miR-200, miR-203, miR-205, miR-206, miR-218, miR-324, miR-363, miR-375, miR-451, miR-489, miR-491, miR-506, miR-519, miR-639, and let-7d were correlated with lower survival and metastasis (Supplementary Table 1). Then, the role of these miRNAs was investigated individually as either pro-metastatic (*n* = 29) or anti-metastatic (*n* = 28). From the manuscripts that showed HR and significant statistical correlation between microRNA and outcomes for HNSCC, elevated expressions of 22 miRNAs were associated with lower survival rates, whilst decreased expressions of 19 miRNAs were associated with poor outcomes (Table 1). These miRNAs are playing different hallmark in head and neck cancer progression to metastasis (Figure 2)

**Figure 3:**
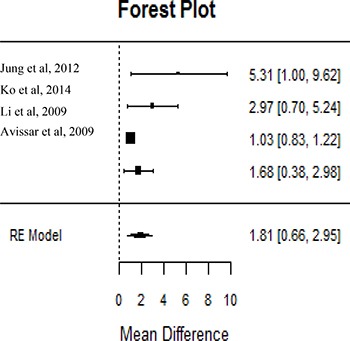
MiR21 level was predictor of poor prognosis in HNSCC Studies evaluating hazard ratios showed high miR21 expression associated with poor patient's outcomes. Survival data are reported as overall survival.

Six of the most significantly deregulated miRNAs: miR-21, miR-34, miR-93, miR-155, miR-196, miR-211, were reported by the majority of the datasets. However, due to the relatively small sample size, heterogeneous methodologies, distinct tumor location, and assorted clinical stages, only miR-21 was included in the meta-analysis (Figure 3). The parameters mentioned above were necessary to perform the meta-analysis in order to avoid inconsistency of biological conclusions. After eliminating heterogeneity among the studies involving miR-21, four manuscripts [15, 23–25] with 456 subjects were included in meta-analysis, with qRT-PCR being used for most samples (75%). The studied populations belonged to United States (*n* = 186, 40.8%), China (*n* =103, 22.6%) and South Korea (*n* =167, 36.6%). For this analysis, miR-21 level confirmed to be significantly associated with poor prognosis in HNSCC (Pooled HR = 1.81–95% CI: 0.66–2.95, *P* < 0.005).

### Target prediction and enrichment analysis

**Figure 4:**
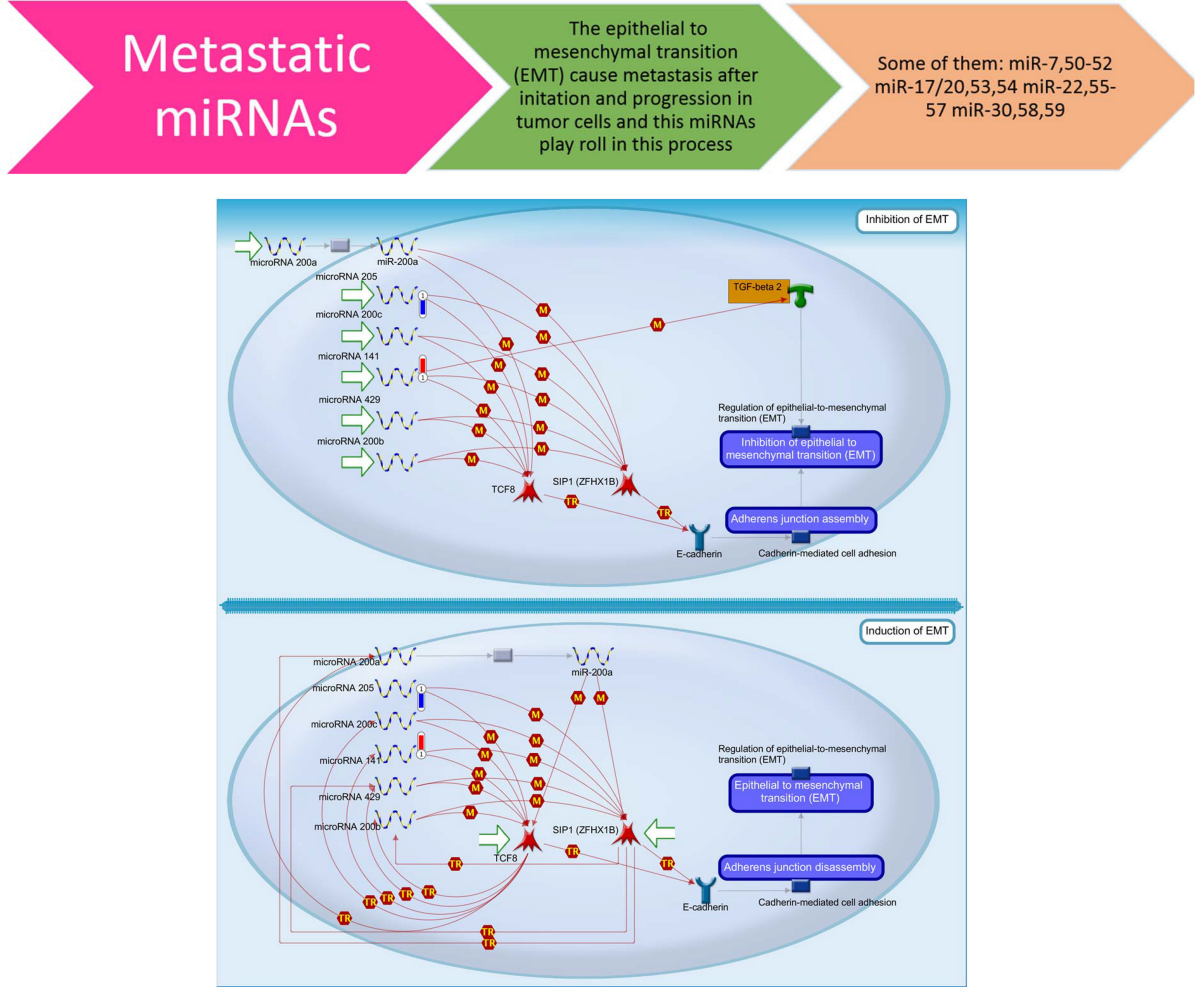
MicroRNA related with head and neck cancer prognosis that are linked with epithelial to mesenchymal (EMT) process during tumor invasion A characteristic of metastasizing cells is their transition from an epithelial to a mesenchymal state, a process known as EMT. Many microRNAs are involved in regulating EMT in HNSCC. For example: the MIR200 family, shown in the diagram, is a group that is well known to be powerful negative regulators of this process by mediating the effects of TGF-β and other EMT regulators. Decreased expression of these microRNA leads to the down-regulation of E-cadherin, a cell adhesion molecule, causing the disassembly of the adherent junctions, and subsequently induces EMT, which causes cells to gain motility and invade other tissues.

To elucidate the underlying mechanisms of these miRNA in the prognosis of patients with HNSCC, the 53 miRNAs were used as seed for network growth in Gene Go Metacore^TM^. We identified four networks (FDR < 10^–30^ and Z-score > ^90^), which were enriched for metabolic processes, regulation of cell proliferation, response to hormones and regulation of cell plasticity, in particular epithelial to mesenchymal transition (EMT) (Figure 4, Table 2).

**Table 2:** Molecular processes of the miRNAs associated with poor outcomes in HNSCC

Network	GO processes	Total nodes	Seed nodes	*p*-Value	z Score	g Score
miR-205-5p,miR-21-5p,miR-195-3p,miR-34a-3p,miR-21-3p	positive regulation of macromolecule metabolic process (89.3%; 1.356e-16), positive regulation of protein metabolic process (75.0%; 2.924e-16), positive regulation of signal transduction (71.4%; 9.780e-16), positive regulation of response to stimulus (78.6%; 1.709e-15), positive regulation of cellular metabolic process (85.7%; 6.069e-15)	50	23	1.020E-61	130.32	130.32
miR-31-5p,miR-143-3p,miR-203-3p,miR-17-5p,miR-211-3p	positive regulation of multicellular organismal process (60.6%; 4.452e-13), histone H4 deacetylation (15.2%; 3.744e-11), positive regulation of cellular process (81.8%; 1.796e-10), regulation of cell proliferation (54.5%; 2.356e-10), single-multicellular organism process (87.9%; 2.828e-10)	50	17	4.280E-43	97.26	97.26
miR-143-3p,miR-206-3p,miR-96-5p,miR-195-5p,miR-152-3p	regulation of transcription from RNA polymerase II promoter (71.9%; 5.460e-16), positive regulation of macromolecule metabolic process (78.1%; 6.802e-14), positive regulation of cellular metabolic process (78.1%; 1.002e-13), negative regulation of transcription, DNA-templated (56.2%; 3.559e-13), negative regulation of nucleic acid-templated transcription (56.2%; 5.897e-13)	50	17	6.480E-43	96.28	96.28
miR-205-5p,miR-34a-3p,microRNA 18a,miR-491-5p,microRNA 31	response to endogenous stimulus (62.2%; 7.458e-13), regulation of transcription from RNA polymerase II promoter (59.5%; 8.950e-13), cellular response to chemical stimulus (70.3%; 1.448e-12), cellular response to organic substance (64.9%; 2.931e-12), response to organic substance (70.3%; 2.105e-11)	50	16	6.270E-40	90.6	90.6

**Figure 5:**
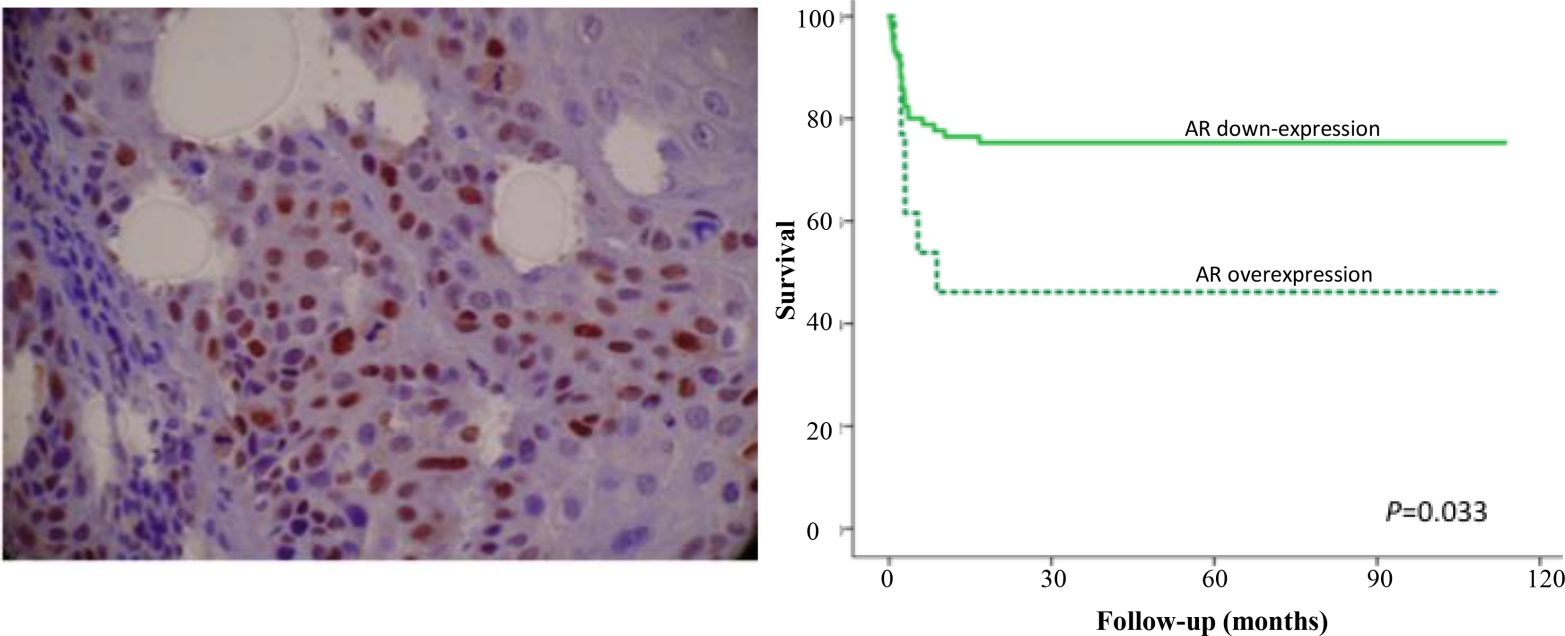
Representative immunohistochemical staining for androgen receptor in head and neck cancer The nuclear immunoreactivity for androgen receptor (AR) was easily identified. Original magnification: 200x. Survival curves analysis according to the Kaplan–Meier method showing that patients with positive expression of AR had shorter survival rate in comparison with negative immunostaining (log-rank test, *P* < 0.05).

One of these specific network involved with response to hormone (androgen receptor-transcription factor) showed that the AR is directly or indirectly regulated by 16 miRNAs (out of the 53, 30.1%), suggesting that this protein could have a central role and may be involved with tumor progression to metastasis. 30.0% of the all the microRNA studied in head and neck cancer to determine patient's prognosis was enriched in one network to regulate AR gene expression. However, in order to analyze whether this alteration affected the translational level, as a proof of concept, we explored the potential involvement of AR protein expression in a cohort of HNSCC patients with 10 years of follow-up. Typically, these patients relapse in 2 years. Among 100 patients analyzed, 23 patients (23.0%) had recurrence, 28 patients (28.0%) had distant metastasis, and 50 patients (50.0%) died. 65 samples from 85 cases that were positive for AR had statistically worse disease-free survival probability compared with 15 patients whose tumors down-expressed AR (log-rank test, *P* < 0.05) (Figure 5).

**Figure 6:**
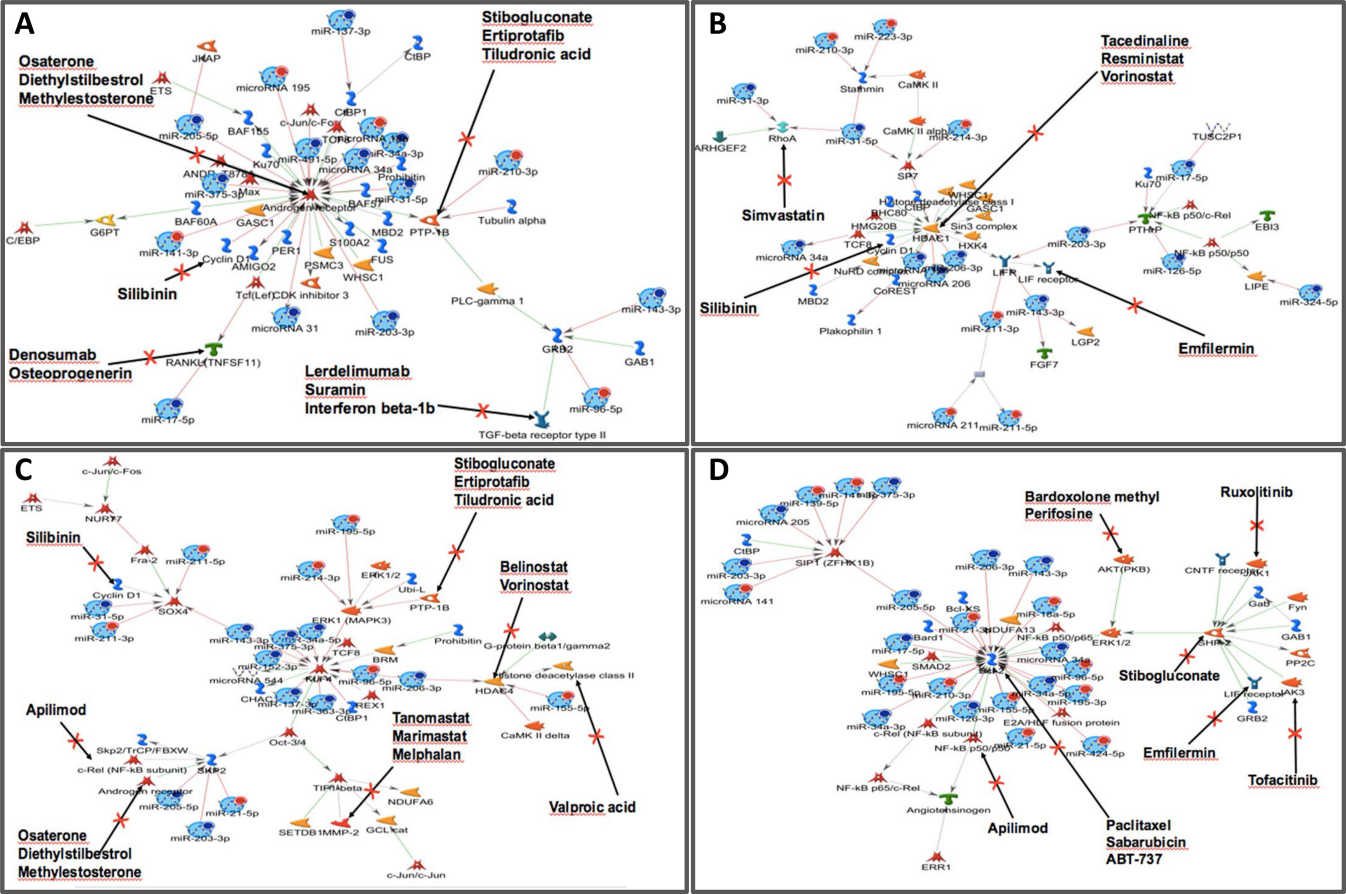
Regulatory network of selected miRNAs and their differentially expressed target genes associated with poor prognosis in HNSCC Thirty drugs interfere with four main representative networks in HNSCC. (Metacore^TM^) and they are able to act in 32 proteins. These proteins are targeted by the drugs include AR (Osaterone, Diethylstilbestrol and Methylestosterone) and BCL2 (ABT-737, Paclitaxel and Sabarubicin). Specifically for AR, the validated targets included 16 miRNAs involved with direct interaction with AR (arrows directly pointing to AR) or indirect involvement with the AR gene (arrows pointed for another genes with indirectly regulation of AR expression). Graphs were extracted from Metacore^TM^, Thompson Reuters (https://portal.genego.com).

Finally, considering that these 53 miRNAs are related to prognosis, we searched for drugs interfering with the networks using Metacore^TM^. We found 30 drugs acting in 32 proteins in the four networks identified (Supplementary Table 3, Figure 6). Proteins targeted by the drugs include AR (Osaterone, Diethylstilbestrol and Methylestosterone), TGF-B (Lerdelimumab, Suramin, and Interferon beta), PTP-1B (Ertiprotafib, Stibogluconate and Tiludronic acid), TNFSF11 (Denosumab and Osteoprogenerin), CCND1 (Silibinin) (Figure 6A); HDAC1 (Tacedinaline, Resministat, and Vorinostat), RhoA (Simvastatin), LIFR (Emfilermin) (Figure 6B); HDAC4 (Belinostat and Vorinostat), MMP2 (Tanomastat, Marimastat and Melphalan), c-Rel (Apilimod), Histone deacetylase (Valproic acid) (Figure 6C); BCL2 (ABT-737, Paclitaxel, and Sabarubicin), AKT (Bardoxolone methyl and Perifosine), PTP-1B (Stibogluconate), JAK1 (Ruxolitinib) and JAK 3 (Tofacitinib) (Figure 6D). The complete list of potential drugs acting in proteins regulated by the microRNAs in head and neck cancer and their function is presented in Supplementary Table 3.

## DISCUSSION

Despite advances in surgery and chemoradiation approaches for HNSCC, only limited improvement in survival rates have been achieved over the last decades, stressing a urgent need for innovative therapeutic approaches with greater efficacy, particularly for recurrent and metastatic tumors. In this context, miRNAs have emerged to play a determinant regulatory role in carcinogenesis and to fulfill a potential utility as biomarkers, which could yield innovative therapies for HNSCC. In this paper, we explored the current knowledge regarding miRNAs and HNSCC, in order to develop a strategy of identifying potential new biomarkers for prognostic and therapeutic applications. We did a comprehensive systematic review of the published literature searching for studies involving miRNAs and HNSCC outcomes. Then, we associated the biological process with potential drugs that can interfere with miRNA regulation. To the best of our knowledge, this is the first extensive report to bring to the scientific community the insight to develop faster system to target miRNA (with drugs already available in the market).

Our review showed elevated expression of miR-7, miR-9, miR-15, miR-18, miR-19, miR-21, miR-23, miR-24, miR-93, miR-96, miR-99, miR-130, miR-139, miR-141, miR-155, miR-181, miR-195, miR-196, miR-210, miR-211, miR-214, miR-222, miR-296, miR-302, miR-331, miR-345, and miR-424 were associated with poor prognosis in HNSCC. Conversely, decreased expressions of miR-17, miR-26, miR-29, miR-31, miR-34, miR-125, miR-126, miR-137, miR-138, miR-143, miR-152, miR-200, miR-203, miR-205, miR-206, miR-218, miR-324, miR-363, miR-375, miR-451, miR-489, miR-491, miR-506, miR-519, miR-639, and let-7d were correlated with lower survival and metastasis. The expression patterns of these miRNAs were correlated with clinical stage, lymph node metastasis, and patient survival, indicating that they can act as prognostic predictors in HNSCC. Although these miRNAs showed association with patients’ outcomes, their roles during the metastatic process are still under investigation. Among all the miRNAs, miR-21 has been studied more frequently as a prognostic marker of HNSCC in recent years. Higher expression levels of miR-21 were correlated with advanced clinical stage, poor differentiation, and lymph node metastasis in tongue cancer [15, 17, 18, 24, 25, 48]. However, many of the studies reporting miR-21 as a prognostic factor were excluded in our meta-analysis due to the absence of HR or an inadequate/unrelated report of prognostic factors. In other words, conclusions based on limited number of patient samples from different sub-locations of HNSCC were excluded, as they lead to more heterogeneous data and reduced statistical power. Then, because of this heterogeneity, only four studies were included in the meta-analysis [15, 23–25], which showed elevated miR-21 levels as a predictor of poor prognosis in HNSCC. Although statistically significant, the conclusion was not strong, with a pooled HR of 1.81 (95% CI: 0.66–2.95, *P* < 0.005). Another point to be considered is the different expression profiling, such as ISH or PCR, with different normalizing strategies. Thus, the extrapolation of the results to a broader sense and generalization of the findings necessitates further investigation. Similarly, miR-31, miR-17/20a, miR-125b, miR-155, miR-181, miR-375, miR-491-5p, miR-205, and let-7d were found to be associated with lymph node metastasis and poor oral squamous cell carcinoma (OSCC) patient survival [16, 17–20, 24, 38, 49], but the literature does not have enough studies involving these microRNAs in head and neck that justify a meta-analysis.

The most promising application of miRNAs might lie in estimation of outcome and also in the modification of response in known and well established anti-tumour therapies, such as radiation and chemotherapy. For example, alterations in miRNA expression profiles could provide information about sensitivity or resistance of certain tumour types to different treatments before starting any therapy (‘response prediction’); alternatively, or in addition, changes in expression during a therapy might offer a tool for control of success of treatment (‘response control’) [50]. Currently, cisplatin-based chemotherapy or concurrent radiochemotherapy is still the first choice to treat the advanced stage head and neck cancers, in particular, the unresectable tumours [51–54]. Unfortunately, innate and acquired resistance to chemotherapy agent greatly limited its effectiveness and often led to treatment failure in these patients. Six miRNAs showed an association with response to chemotherapy in HNSCC. Deregulation of the miR-222-ABCG2 in tongue squamous cells carcinoma was correlated with cisplatin resistance and enhanced migratory/invasive potential [51]. miR-23, miR-10 and miR-203 also showed to induce survival proteins and cisplatin chemoresistance in HNSCC [52–54]. Experiments using nasopharyngeal carcinoma cells *in vitro* and animal model showed that miR-604 and miR-1204 expression enhances the sensitivity to paclitaxel [55, 56]. Therefore, miR-10, miR-23, miR-222 and miR-203 were correlated with cisplatin resistance and enhanced migratory/invasive potential during head and neck cancer progression [51–54]. On the other hand, miR-604 and miR-1204 expression enhances the sensitivity to paclitaxel [55, 56]. These microRNAs play important roles in sensitization of tumor cells to different classes of anti-cancer drugs.

However, the survival outcomes for patients with advanced HNSCC remain poor over the last several decades. This implies that after surgery, chemotherapy may not be a proper choice, as tumors of this region are relatively resistant to cytotoxic drugs [6]. A modest progress in the patient's outcomes can be observed after radiotherapy as opposed to chemotherapy [5]. Consequently, clinicians and researchers’ expectations are focused on targeted therapy, where microRNAs seem to be the most promising tool [4]. In the last 20 years, miRNAs became new players on the scene of cancer science. Since then, extensive investigations have been performed with a hope to find a new prognostic tool to understand the basis of molecular carcinogenesis. The ability to manipulate miRNAs expression and function by local and systemic delivery of miRNA inhibitors (anti-miRNA oligonucleotides or miRNA sponges [57, 58]) or miRNA mimics [58] has recently gained interest as novel therapeutic approach. The advantage of miRNA based cancer therapy lies in the ability of miRNAs to concurrently target multiple effectors of pathways involved in cell proliferation, differentiation, and survival [57]. Accordingly, several studies in preclinical and animal models, using strategies to suppress the function of oncogenic miRNA and/or to restore the tumor suppressive miRNAs, have reported significant inhibition of aggressive phenotypes in HNSCC [7]. For example, inhibiting miR-21 by anti-miRNA oligonucleotides has been shown to inhibit survival, anchorage-independent growth [59], and invasion in oral cancer cell lines [60]. Likewise, restoration of miR-99a level by miR mimic transfection markedly suppressed proliferation and induced apoptosis of oral cancer cells [61]. Recently, nanoparticle-based delivery of miRNAs was proposed as a promising approach for the treatment of HNSCC [62]. However, more in-depth studies are necessary to better identify effective delivery system for optimal uptake and to minimize degradation of miRNA based drugs in the *in vivo* condition.

Miravirsen is the first miRNA-targeted drug to receive Investigational New Drug (IND) acceptance from the FDA, although it is not yet for treatment of cancers [63]. Other miRNA-based therapies are still at the stage of preclinical or early clinical trials and their utility is awaiting to be proven beyond patent documentations [64]. Some of the miRNAs are fairly good candidates for being included into therapies, however, this has not been clinically verified yet. One of the most important targets for new anticancer therapies is the programmed cell death pathway [65]. Neoplastic cells usually lose the ability to undergo apoptosis. Effective pro-apoptotic agents would increase apoptosis as normal cell function and directly or indirectly decrease tumor expansion [65]. Yan et al. have shown that miR-99a mimics markedly induced apoptosis in oral cell line and inhibited cell proliferation [61]. Further, restoration of miR-100 to the HNSCC cell lines enhanced apoptosis and thus suppressed cell proliferation and migration [60]. These are only some recognized examples, since a considerable fraction of functionally investigated miRNAs in HNSCCs may be linked to modifications of apoptotic and cell death signaling pathways and potentially comprise a clear objective for further testing to find new therapeutic factors. Sixteen miRNAs from our meta-analysis regulate AR expression. AR is a DNA binding transcription factor that translocates to the nucleus after binding to androgenic hormones, testosterone, or dihydrotestoterone [66]. AR regulates the transcription of multiple effector genes through direct DNA binding or interaction with other transcription factors, leading to increased cell growth, differentiation, and survival [67]. AR signaling is an important oncogenic driver in several tumor types, including HNSCC, prostate and a subset of breast cancers [68–70]. Due to its role in cancer progression, several drugs have been proposed to target AR as an alternative treatment [71]. We found 11 drugs targeting the AR network, with Osaterone, Diethylstilbestrol and Methylestosterone targeting specifically AR.

## CONCLUSIONS

Several miRNAs are established to play critical roles in the initiation and progression of HNSCC by functioning either as oncogenes or as tumor suppressors. Specific miRNA signatures identified from tumor specimens, serum/plasma, or saliva from patients may have a clinical relevance to serve for diagnosis, prognosis, and/or therapeutic targets in HNSCC. The current evidence suggests that miRNAs have potential prognostic value to serve as screening tools for clinical practice in HNSCC follow-up and treatment. However, larger-scale studies are required to improve the accuracy and explore the most effective combination to target miRNAs.

## SUPPLEMENTARY MATERIALS TABLES






