# Supplementary material for: Meta-analysis of microRNAs expression in head and neck cancer: uncovering association with outcome and mechanisms
Source: Oncotarget. 2017 Jul 13;8(33):55511–24. doi: 10.18632/oncotarget.19224 (PMC5589676; doi:10.18632/oncotarget.19224)
Supplement: Supplementary file 1 [file oncotarget-08-55511-s001.pdf]

Meta-analysis of microRNAs expression in head and neck cancer: uncovering association with outcome and mechanisms

SUPPLEMENTARY MATERIALS

Supplementary Table 1: Clinicopathological variables of patients with head and neck squamous cell carcinoma

Variable	Category	Paraffin-embedded samples *n* (%)
Age	< 50 year	34 (34)
	≥ 50 year	66 (66)
Gender	Male	81 (81)
	Female	19 (19)
Smoking habit	No	77 (89.5)
	Yes	9 (10.5)
Alcohol consumption	No	19 (22.1)
	Yes	67 (77.9)
T category	T1 + T2	33 (35.5)
	T3 + T4	60 (64.5)
Nodal status	N0	46 (49.5)
	N+	47 (50.5)
Recurrence or metastasis	No	72 (72)
	Yes	28 (28)
Survival	Alive	62 (62)
	Dead	38 (38)

Supplementary Table 2: Characteristics and data from the included studies about microRNAs and prognostic factor in head and neck squamous cell carcinoma. See Supplementary_Table_2

Supplementary Table 3: List of potential drugs acting in proteins in significant networks related with the miRNAs in HNSCC. See Supplementary_Table_3
